# Dilated cardiomyopathy impairs mitochondrial biogenesis and promotes inflammation in an age- and sex-dependent manner

**DOI:** 10.18632/aging.202283

**Published:** 2020-12-02

**Authors:** Maria Luisa Barcena, Sofya Pozdniakova, Natalie Haritonow, Pavelas Breiter, Anja A. Kühl, Hendrik Milting, Istvan Baczko, Yury Ladilov, Vera Regitz-Zagrosek

**Affiliations:** 1 Clinic for Geriatrics, Charité University Hospital, Berlin, Germany; 2 DZHK (German Centre for Cardiovascular Research), Berlin Partner Site, Berlin, Germany; 3 Climate and Health Program (CLIMA), Barcelona Institute for Global Health (ISGlobal), Barcelona, Spain; 4 Charité, Universitätsmedizin Berlin, Corporate Member of Freie Universität Berlin, Humboldt, Universität zu Berlin, Berlin Institute of Health, iPATH Berlin-Immunopathology for Experimental Models, Berlin, Germany; 5 Erich and Hanna Klessmann Institute, Heart and Diabetes Centre NRW, University Hospital of the Ruhr-University Bochum, Bad Oeynhausen, Germany; 6 Department of Pharmacology and Pharmacotherapy, Interdisciplinary Excellence Centre, University of Szeged, Szeged, Hungary; 7 Institute for Gender in Medicine, Center for Cardiovascular Research, Charité University Hospital, Berlin, Germany; 8 Department of Cardiology, University Hospital Zürich, University of Zürich, Zürich, Switzerland

**Keywords:** dilated cardiomyopathy, inflammation, mitochondrial proteins, sex differences, aging

## Abstract

Dilated cardiomyopathy (DCM) belongs to the myocardial diseases associated with a severe impairment of cardiac function, but the question of how sex and age affect this pathology has not been fully explored. Impaired energy homeostasis, mitochondrial dysfunction, and systemic inflammation are well-described phenomena associated with aging. In this study, we investigated if DCM affects these phenomena in a sex- and age-related manner.

We analyzed the expression of mitochondrial and antioxidant proteins and the inflammatory state in DCM heart tissue from younger and older women and men.

A significant downregulation of Sirt1 expression was detected in older DCM patients. Sex-related differences were observed in the phosphorylation of AMPK that only appeared in older males with DCM, possibly due to an alternative Sirt1 regulation mechanism. Furthermore, reduced expression of several mitochondrial proteins (TOM40, TIM23, Sirt3, and SOD2) and genes (*cox1*, *nd4*) was only detected in old DCM patients, suggesting that age has a greater effect than DCM on these alterations. Finally, an increased expression of inflammatory markers in older, failing hearts, with a stronger pro-inflammatory response in men, was observed. Together, these findings indicate that age- and sex-related increased inflammation and disturbance of mitochondrial homeostasis occurs in male individuals with DCM.

## INTRODUCTION

Dilated cardiomyopathy (DCM) is a non-ischemic heart muscle disease with structural and functional myocardial abnormalities characterized by dilation of the ventricular chamber and impaired contraction [1]. Myocardial damage triggers inflammation followed by the recruitment of immune cells to the injured site and the release of cytokines [2, 3].

Furthermore, aging is considered to be a risk factor for cardiovascular diseases and to have a negative impact on ventricular function [4–7]. On the molecular level, aging is accompanied by a disturbance in energy homeostasis, mitochondrial dysfunction, and increased inflammation [4]. Importantly, sex differences in the mitochondrial function in several pathologies, including cardiovascular diseases, have been demonstrated [8]. Mitochondrial biogenesis and respiration are regulated by PGC-1α, a transcriptional coactivator, and its downregulation has been shown to lead to hypertrophy and heart failure [9–11]. Furthermore, the reduced activity of PGC-1α itself, as well as its modulators, especially AMP-activated kinase (AMPK) among others, has been associated with aging [12]. A decline in AMPK activity, a crucial regulator of energy metabolic homeostasis, has been shown in older subjects, and increasing this AMPK activity may extend lifespans [13]. Sirtuins (Sirt), a conserved family of global metabolic regulators with NAD^+^-dependent deacetylase activity [14], and Sirt1 (an evolutionarily conserved AMPK partner) in particular [15, 16], have been widely reported to protect against age-associated diseases and therefore, to increase health span and life span [17–21]. Consistent reduction of NAD^+^ levels in older mice is accompanied by a decrease in Sirt1 activity, while its genetic or pharmacological restoration promotes longevity [18, 22, 23].

Among other factors, AMPK and Sirt1 play essential roles in mitochondrial biology. Particularly, AMPK and Sirt1 promote PGC-1α activity and, thus, mitochondrial biogenesis. Furthermore, AMPK controls mitochondrial clearance, i.e., mitophagy [24] and a decline in the AMPK activity leads to impaired mitophagy, accumulation of dysfunctional mitochondria, and ROS formation, which may trigger inflammation- and aging-related diseases [4, 25, 26]. Additionally, the release of mtDNA from damaged mitochondria into the cytosol may lead to an inflammatory response, via Toll-like receptors and STING-dependent inflammasome activation [25]. Similarly, emerging data have suggested that Sirt1 may play an anti-inflammatory role [27–29].

In addition to Sirt1, Sirt3 is another sirtuin playing an essential role in mitochondrial biology. Sirt3 is the main mitochondrial-localized deacetylase that maintains the activity of numerous mitochondrial enzymes, e.g. SOD2, and thus, supports metabolic and redox balance in mitochondria [30]. Both we and others have shown a decline in Sirt3 expression in older human myocardium [31, 32]. It is important to note that an anti-inflammatory role of mitochondria-localized Sirt3 has been reported [33, 34].

Sex is another potential risk factor associated with cardiovascular diseases. Men have an increased incidence and severity of atherosclerosis, myocardial infarction, heart failure, and DCM [35–37], whereas women with DCM have better chances of survival than men [38]. Heart failure is associated with cardiomyocyte hypertrophy, apoptosis, inflammation, and interstitial fibrosis, which all occur in a sex-specific manner [39]. Estrogen (E2) seems to play a protective role, as a decline in its levels is associated with deleterious left ventricle remodeling and cardiac dysfunction [40].

Altogether, sex and age have significant impacts on cardiomyopathy, however, there is still a large gap in the research about signaling in heart diseases dependent on age or sex. Here, we investigated age- and sex-related alterations in the expression of metabolic regulators, i.e., AMPK and Sirt1, mitochondrial biogenesis, and inflammation parameters in patients with DCM. The analyses revealed a decreased Sirt1 and Sirt3 expression in older DCM patients in both sexes, whereas the activity of AMPK was increased only in men. Sex differences were also found in mitochondrial antioxidant capacity, e.g., SOD2. DCM in the hearts of older patients was associated with a reduced expression of mitochondrial proteins and increased inflammation, which were both sex dependent and independent.

## RESULTS

### DCM-related Sirt1 and AMPK alterations in older patients

Sirt1 and AMPK are key regulators of metabolic pathways. Previously, we observed a decreased expression of Sirt1 and AMPK in older individuals [32]; however, age- and sex-related differences in patients with DCM remained unexplored. Therefore, in the present study, the expression of Sirt1, AMPK, and pAMPK in control (non-diseased) and diseased human cardiac tissue from young and old male and females was analyzed.

**Figure 1:**
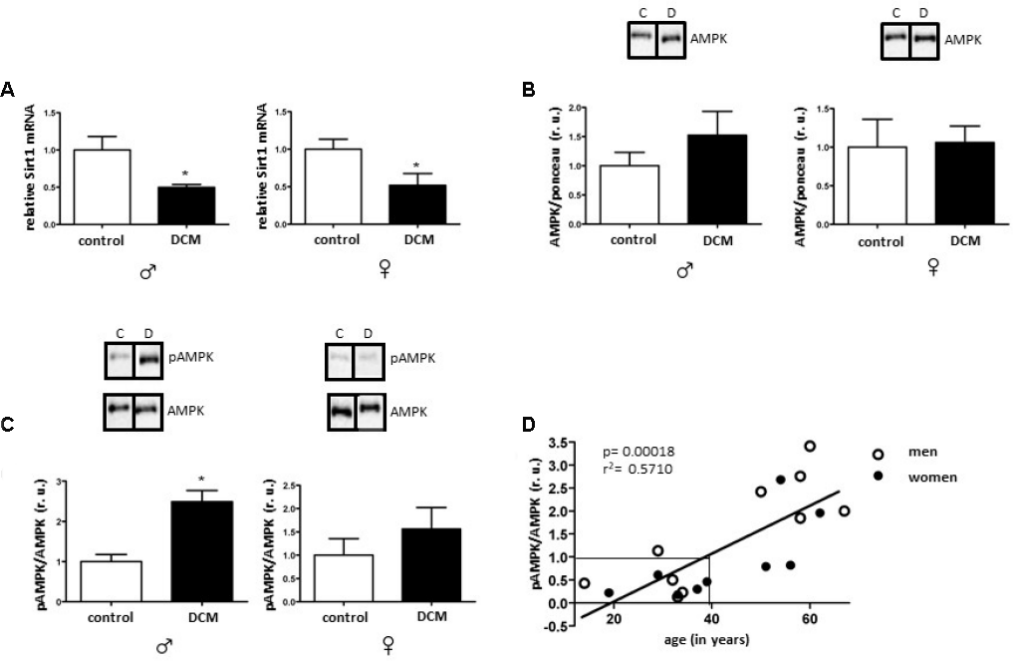
**Effects of DCM on Sirt1 expression and AMPK phosphorylation in older patients.** Expression analysis of Sirt1 mRNA (**A**), total AMPK (**B**) and phosphorylated AMPK (Thr172) (**C**) performed with human cardiac tissue lysates from old control (non-diseased) and DCM men (♂) and women (♀). Data are means ± SEM (n= 5). Representative imaging of western blot analysis; the lanes were run in two gels. All data were normalized to the corresponding control and expressed in relative units (r.u.). (**D**) Linear regression analysis between pAMPK/AMPK ratio (dependent variable) and age (explanatory variable) was performed with function lm() in R. pAMPK/AMPK values were obtained from old and young male and female hearts diagnosed with DCM.

In older individuals, Sirt1 expression was significantly decreased in the DCM group when compared to the control (Figure 1A). Both Sirt1 and AMPK share many common target molecules and interact with each other [15]. To test whether the Sirt1 downregulation is associated with the alteration of AMPK activity (indirectly highlighted by the phosphorylation rate), the pAMPK/AMPK ratio was analyzed. A significant increase in AMPK phosphorylation in older men with DCM, but not in women, in the presence of unchanged total AMPK content, was observed (Figure 1B, 1C). Surprisingly, the pAMPK/AMPK ratio was markedly reduced in younger individuals (Supplementary Figure 1C). The linear regression analysis revealed a significant dependence of the DCM-related alteration in the AMPK phosphorylation on age, with the threshold appearing at 40 years (Figure 1D). Therefore, DCM seems to have an opposite effect on AMPK phosphorylation in younger (downregulation) and older (upregulation) patients (Figure 1D).

No significant changes in Sirt1 or in AMPK expression in younger individuals with DCM were found (Supplementary Figure 1A, 1B).

### DCM-related expression of mitochondrial and anti-oxidative enzymes in older patients

Disturbance of mitochondrial homeostasis, e.g., biogenesis, is a hallmark and a trigger of heart failure [41]. Therefore, markers of mitochondrial biogenesis were analyzed in cardiac tissue.

PGC-1α is a key transcription co-activator involved in mitochondrial biogenesis and a direct target of Sirt1 [42]. It has been shown that downregulation of Sirt1 may impair PGC-1α activity [42], and, in turn, mitochondrial biogenesis [43].

**Figure 2:**
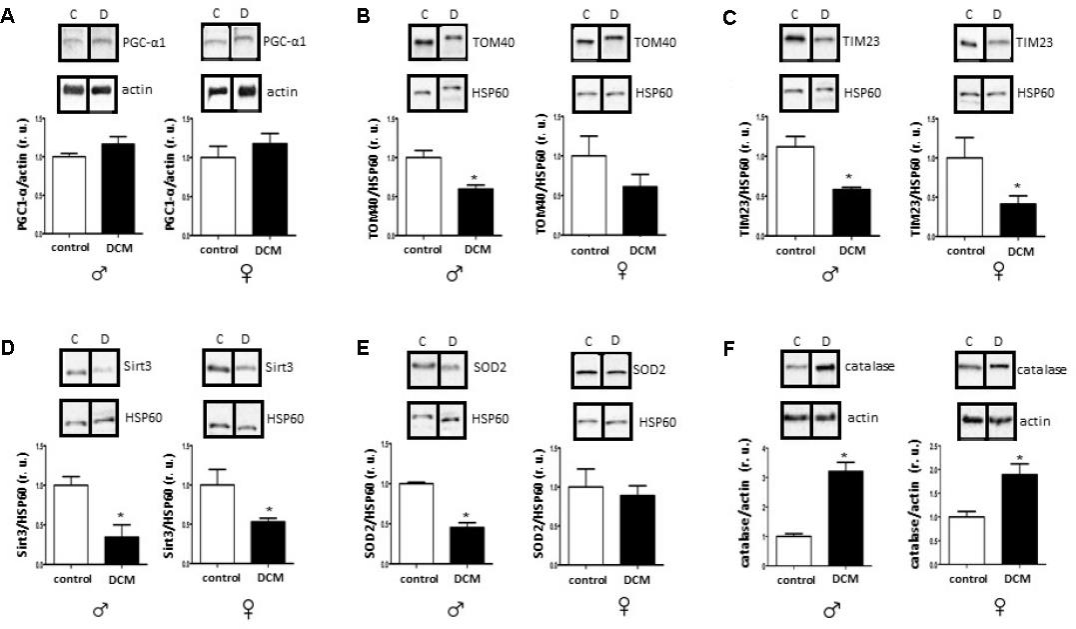
**DCM-related alterations in expression of mitochondrial proteins and anti-oxidative enzymes in older patients.** Western blot analysis of PGC1-α (**A**), TOM40 (**B**), TIM23 (**C**), Sirt3 (**D**), SOD2 (**E**) and catalase (**F**) expression performed with human cardiac tissue lysates from old control (non-diseased) or DCM men (**♂**) and women (**♀**). Representative imaging of western blot analysis; the lanes were run in two gels. All data were normalized to the corresponding control and expressed in relative units (r.u.). Data are means ± SEM (n= 5).

**Figure 3:**
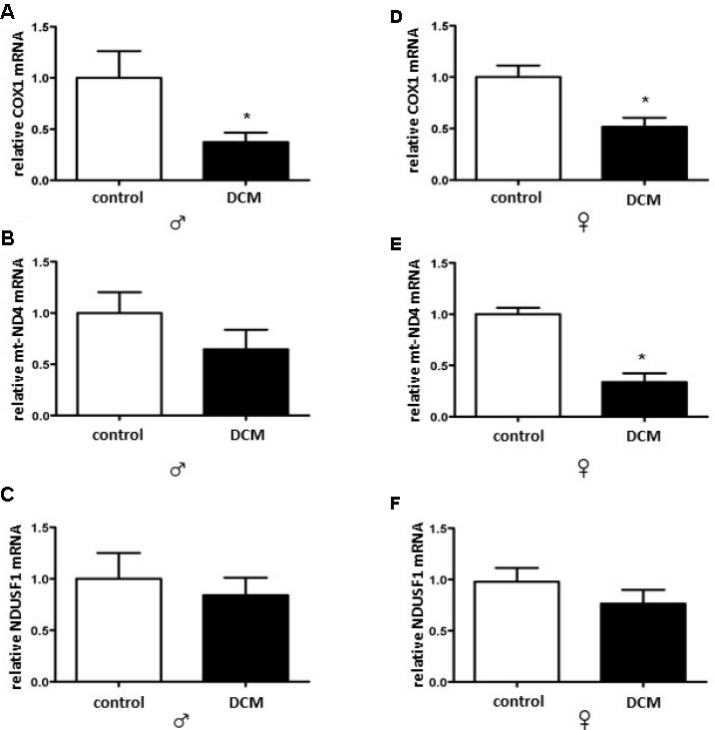
**DCM-related alterations in expression of mitochondrial genes in older patients.** Real-time PCR analysis for the mitochondrial-encoded genes *cox1* (**A**, **B**) and *mt-nda* (**C**, **D**) as well as the nuclear-encoded genes *ndusf1* (**E**, **F**), performed with human cardiac tissue lysates from old control (non-diseased) or DCM men (♂) and women (♀). Data are shown as the mean ± SEM (n= 5).

In the present study, the protein level of PGC-1α was affected neither in an age- nor in a sex-specific manner (Figure 2A and Supplementary Figure 2A). Nevertheless, the expression of several mitochondrial proteins (TOM40, TIM23, and Sirt3), as well as mRNA (*cox1*, mt-*nd4*) was markedly reduced in older but not in younger DCM patients (Figures 2, 3, Supplementary Figure 2 and Supplementary Figure 3, respectively). Of note is the significant reduction of the expression of a key anti-oxidative mitochondrial enzyme SOD2 solely in older male DCM patients (Figure 2E). In contrast, the expression of cytosolic anti-oxidative enzyme catalase was significantly upregulated in older DCM individuals of both sexes (Figure 2F). Taken together, our data suggest a disturbance in mitochondrial biogenesis and anti-oxidative defense in hearts with DCM.

### DCM-related pro-inflammatory state alterations in older patients

The cardiac pro-inflammatory state is expanded during aging processes [44]. Downregulation of Sirt1 and AMPK, along with mitochondrial dysfunction, may significantly contribute to the initiation and perpetuation of inflammation in the heart [45, 46]. Thus, we examined the data for alterations in the Sirt1 and AMPK signaling and mitochondrial homeostasis that were accompanied by a pro-inflammatory response in older DCM patients.

**Figure 4:**
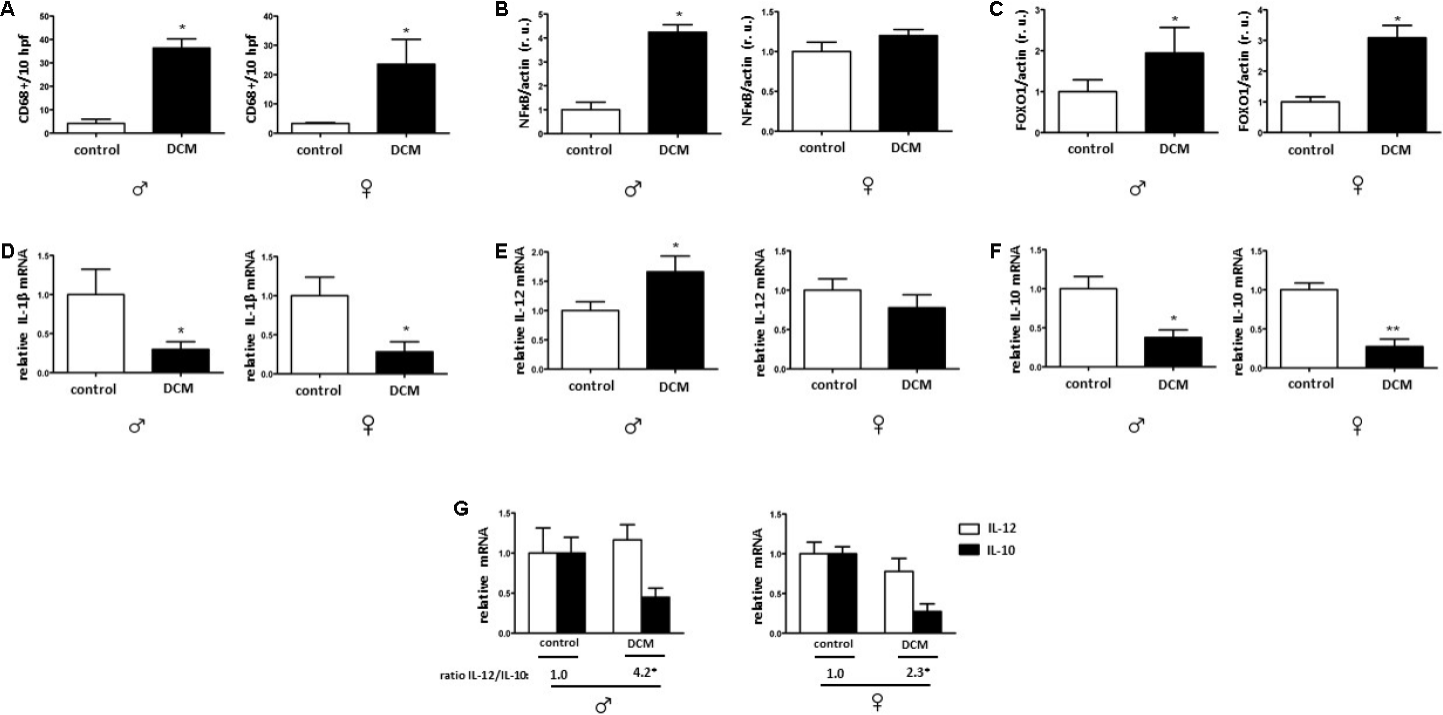
**DCM effects on the pro-inflammatory state.** Immunohistological analysis of CD-68 immuno-reactive cells (**A**). Western blot analysis of NF-κB (**B**), FOXO1 (**C**) as well as the real-time PCR analysis of IL-1β (**D**), IL-12 (**E**), and IL-10 (**F**) mRNA expression performed with human cardiac tissue lysates from old control (non-diseased) or DCM men (**♂**) and women (**♀**). (**G**) Ratio of IL-12/IL-10. Representative imaging of western blot analysis. All data were normalized to the corresponding control and expressed in relative units (r.u.). Data are shown as the mean ± SEM (n= 5).

The number of cardiac CD68 immune-reactive cells was markedly increased in older hearts with DCM when compared to the older control hearts (Figure 4A and Supplementary Figure 4). In accordance with this finding, the NF-κB expression was also significantly elevated in older male DCM hearts (Figure 4B). It has been suggested that FOXO1 is involved in the polarization of macrophages [47]. FOXO1 expression was significantly increased in older DCM patients (Figure 4C), further suggesting a DCM-related pro-inflammatory state.

In contrast to these findings, the mRNA of pro-inflammatory cytokines, e. g. TNF-α was not changed in older DCM hearts (data not shown), and the IL-1β was significantly reduced in older DCM hearts in comparison to control hearts (Figure 4D). In contrast, IL-12 mRNA expression was significantly elevated in older male hearts. (Figure 4E). In addition, the mRNA of the anti-inflammatory cytokine IL-10 was reduced in older male and female hearts (Figure 4F). Further analysis of the IL12/IL10 ratio revealed a significantly higher ratio in older male and female DCM hearts (ratio= 4.2 and 2.3) compared to control male or female hearts (1.0). (Figure 4G) Altogether, the pro-inflammatory response in DCM is stronger in older male hearts.

## DISCUSSION

In the current study, we investigated age- and sex-related alterations in metabolic, mitochondrial, and inflammatory markers in DCM patients. The main findings are as follows: 1) In older patients, DCM is associated with significant Sirt1 downregulation in both sexes, while pAMPK/AMPK ratio upregulation only appeared in males; 2) expression of several mitochondrial proteins (TOM40, TIM23, and Sirt3), including a main mitochondrial antioxidant SOD2, and mRNA (*cox1*, *nd4*) is reduced in older but not in younger DCM patients; 3) expression of the inflammatory markers in older hearts with DCM is increased, with a stronger pro-inflammatory response in older males with DCM. Taken together, these findings indicate a disturbance of metabolic sensing, impairment of mitochondrial biogenesis, and increased inflammation in older individuals with DCM in both sex-dependent and -independent manners.

The crucial role of Sirt1 in inflammation, DNA repair, apoptosis, and aging has already been investigated [48, 49]. Its reduced expression and activity have been associated with various pathologies, e.g., diabetes, Alzheimer's disease and coronary artery disease [50–52]. Sirt1 activation by resveratrol tends to decelerate aging processes and the onset of chronic diseases [53–55]. In the present study, the Sirt1 expression was significantly diminished in DCM hearts in older individuals in a sex-independent manner, while no alterations were observed in younger DCM patients. In accordance with our data, consistent reduction of Sirt1 expression was reported in older mice [56] as well as in control hearts of older women. Likewise, Sirt1-deficient mice showed a progressive DCM strongly associated with mitochondrial dysfunction [57]. Furthermore, reduced Sirt1 expression was reported in monocytes of older patients with cardiovascular diseases [52], suggesting that aging directly aggravates cardiac dysfunction via disruption of Sirt1 signaling, promoting mitochondrial dysfunction, apoptosis, and inflammation as a result.

AMPK is a key Sirt1 partner in regulating metabolic activity and inflammation that is activated under metabolic stress [58]. AMPK and SIRT1 may regulate each other and share many common targets [15]. An increasing number of reports emphasize the supporting role of AMPK activity in cardiac metabolic homeostasis [59]. From the other side, persistent AMPK overactivation might be deleterious in some cases, with a chronic AMPK activation appearing to aggravate the pathological damage that strokes and myocardial ischemia cause [13, 60, 61], by, e.g., decreasing fatty acid oxidation [62]. Furthermore, sustained AMPK activation leads to excessive mitochondrial fragmentation and mitophagy, leading to the depletion of functional mitochondria [63]. Here, DCM in older patients was associated with increased phosphorylation of AMPK compared to the control group, whereas in young DCM patients AMPK phosphorylation was markedly reduced. Though diminished AMPK activity in hearts with DCM has been previously observed [61], no age dependence has been demonstrated. In fact, both we and others [13, 32] observed a marked reduction of the cardiac AMPK phosphorylation in older, control individuals. It is tempting to speculate that, with the reduced AMPK phosphorylation in older hearts, additional DCM-related metabolic impairment may trigger the compensatory activation of the AMPK phosphorylation. Fitting in with this view, decreased ATP levels are commonly observed in DCM patients [64].

The disturbance of metabolic homeostasis in older DCM hearts is further highlighted by the reduced mitochondrial biogenesis observed in the present study. Particularly, a reduced Sirt3 expression in older DCM hearts, a main mitochondria-localized deacetylase involved in the regulation of mitochondrial enzymes activity [65, 66], argues for a disturbed mitochondrial function. The pivotal role of Sirt3 in cardiovascular health/diseases is largely accepted and Sirt3 dysfunction is associated with numerous pathologies [67, 68].

Aside from impaired mitochondrial biogenesis and acetylation capacity, numerous mitochondrial abnormalities have been reported in patients with DCM elsewhere [69–71], which may additionally contribute to the mitochondrial dysfunction in older DCM hearts. Mitochondrial dysfunction is typically accompanied by impaired ATP synthesis with enhanced ROS formation [72]. Though we did not measure adenine nucleotide homeostasis in the present study, the enhanced activation of key metabolic sensors, i.e., AMPK, solely in older male DCM hearts suggests the elevation of the AMP/ATP or ADP/ATP ratio. Interestingly, a male-specific downregulation of the main mitochondrial antioxidant enzyme SOD2 was found in older DCM hearts. Altogether, the data suggest that DCM aggravates metabolic and oxidative stress in older hearts in both a sex-dependent and -independent manner.

The results of research conducted thus far suggests that the higher incidence of cardiac diseases, particularly of DCM, in males may be due to the fact that females are protected by higher levels of steroid hormones, 17β-estradiol among others. However, in older, post-menopausal females, the protective effect of 17β-estradiol is lost. In fact, older men have higher 17β-estradiol concentrations in their blood compared to older women [73]. Our recent report [32] showed that cardiac aging is defined by the decline in mitochondrial anti-oxidative defense and a pro-inflammatory shift in females but not in males.

An increased inflammatory response accompanied by decreased mitochondrial function [74, 75] is typical for older hearts.

NFκB promotes inflammation in myocardial tissue in age-related diseases [76]. In accordance with this function, our results showed enhanced NFκB expression in DCM hearts in older males. [27, 77]. Pro-inflammatory mediators are increased in aging under certain physiological conditions [78]. In the present study, the expression of pro-inflammatory factor IL-12 was elevated in older DCM hearts only in males, while a substantial decrease in IL-10 expression was observed in older DCM hearts in a sex-independent manner. A reduced IL-10 expression is a typical fingerprint observed in cardiac aging [32] as well as in aging macrophages [79]; furthermore, IL-10 deficiency promotes a detrimental course of inflammation [80]. The total amount of cardiac macrophages was elevated in DCM patients in this study and accompanied by a shift in the IL-12/IL-10 ratio to IL-12 side, which is a marker of pro-inflammatory macrophages [81].

Our results, in combination with those from other previous studies, suggest an increased inflammatory phenotype in older DCM hearts, which is even more pronounced in males.

In conclusion, the present study revealed that DCM is associated with the downregulation of key metabolic regulator Sirt1, diminished mitochondrial biogenesis and anti-oxidative defense, and an increased inflammatory response in older DCM hearts in both sex-dependent and -independent manners.

## MATERIAL AND METHODS

### Human samples

Human lateral left ventricular (LV) wall tissue from patients with idiopathic end-stage DCM was collected during organ transplantation (men=10 and women=10) and from healthy organ donors (men=16 and women=15). The informed consent from all donors or their legal guardians was obtained. The patients with DCM had ejection fractions (EF) <30%. The tissue was frozen in liquid nitrogen immediately after collection and stored at -80° C. The patients were between 19 and 70 years old, while healthy donors were between 17 and 68 years of age. The control (non-diseased) LV samples were divided into 4 groups of young (17-40 years; male: n=7 and female: n=7) and old (50-68 years; male: n=9 and female: n=8) individuals; DCM samples were divided into 4 groups of young (19-40 years; male: n=5 and female: n=5) and old (50-70 years; male: n=5 and female: n=5) individuals.

For DCM: Sample collection and the experimental protocols were approved by the scientific boards at the Heart and Diabetes Centre (HDZ) NRW (21/2013) and at the Charité – Universitätsmedizin Berlin (EA2/158/16). All research was performed in accordance with the guidelines from the relevant regulatory German authorities.

For healthy donors: The scientific board at the Hungarian Ministry of Health (ETT-TUKEB: 4991-0/2010-1018EKU) approved the sample collection and the experimental protocols. All research was performed in accordance with the German and Hungarian regulatory guidelines.

### RNA extraction and quantitative real-time PCR

Total RNA from cardiac human tissue was homogenized in RNA-Bee (Amsbio, Abbingdon, UK) and the Phenol/Chloroform (Roth, Karlsruhe, Germany) extraction protocol was used for the RNA isolation. The Caliper LabChip bioanalyzer (Agilent Technologies, Ratingen Germany) was used to analyze the purity of the isolated RNA. Quantitative real-time PCR were performed using the Brilliant SYBR Green qPCR master mix (Applied Biosystems, Foster City, CA, USA). The relative amount of target mRNA was determined using the comparative threshold (Ct) method as previously described [34]. The mRNA contents of target genes were normalized to the expression of hypoxanthine phosphoribosyl transferase (HPRT).

### Protein extraction and immunoblotting

LV samples from DCM and control hearts were homogenized in a Laemmli buffer (253mM Tris/HCL pH 6.8, 8% SDS, 40% glycerin, 200mM DTT, 0.4% bromophenol blue). Proteins were quantified using the BCA Assay (Thermo Scientific Pierce Protein Biology, Schwerte, Germany). Equal amounts of total proteins were separated on SDS-polyacrylamide gels and transferred to a nitrocellulose membrane. The membranes were immunoblotted overnight with the following primary antibodies: AMPK (1:2000, Cell Signaling, USA), p-AMPK (1:2000, Thr172, Cell Signaling, USA), PGC-1α (1:1000 Abcam, UK), TOM40 (1:1000, Abcam, UK), TIM 23 (1:5000, BD, USA), Sirt3 (1:1000, Cell Signaling, USA), SOD2 (1:1000, Santa Cruz, USA), catalase (1:1000, Cell Signaling, USA), NFκBp65 (1:200, Santa Cruz, USA) and FOXO1 (1:1000, Cell Signaling, USA). Equal sample loading was confirmed by analysis of actin (1:1000, Santa Cruz, USA), HSP60 (1:1000, Cell Signaling, USA) or Ponceau S staining. Immunoreactive proteins were detected using ECL Plus (GE Healthcare, Buckinghamshire, UK) and quantified with ImageLab (version 5.2.1 build 11, Bio-Rad Laboratories (USA)).

### Immunohistochemistry

For immunohistochemistry, 5 μm cryo-sections of human LV were fixed in formalin for 1 hour at room temperature and subjected to a heat-induced epitope retrieval step prior to incubation with anti-CD68 antibody (clone PGM-1, Agilent Technologies, Santa Clara, CA, USA). The detection was performed by the LSAB method applying the Dako REAL™ Detection System (Agilent Technologies, Santa Clara, CA, USA). Nuclei were counterstained with hematoxylin and mounted on slides with glycerol gelatin (both Merck KGaA, Darmstadt, Germany). Negative controls were performed by omitting the primary antibody. Images were acquired using an AxioImager Z1 microscope (Carl Zeiss MicroImaging, Inc.). Positive cells were quantified in 10 high power fields (hpf) (field of vision in x400 original magnification). All evaluations were performed in a blinded manner.

### Statistical analysis

The data are given as the mean ± SEM. The GraphPad Prism 5 (GraphPad Software, 2003, San Diego, USA) was used for statistical analysis. The data were evaluated using the non-parametric test (Mann-Whitney test for two independent groups). A simple linear regression analysis was performed with function lm() in R. Statistical significance was accepted when p < 0.05.

## Supplementary Material

Supplementary Figures
