# Supplementary material for: Dilated cardiomyopathy impairs mitochondrial biogenesis and promotes inflammation in an age- and sex-dependent manner
Source: Aging (Albany NY). 2020 Dec 2;12(23):24117–33. doi: 10.18632/aging.202283 (PMC7762497; doi:10.18632/aging.202283)
Supplement: Supplementary Figures [file aging-12-202283-s001.pdf]

SUPPLEMENTARY FIGURES

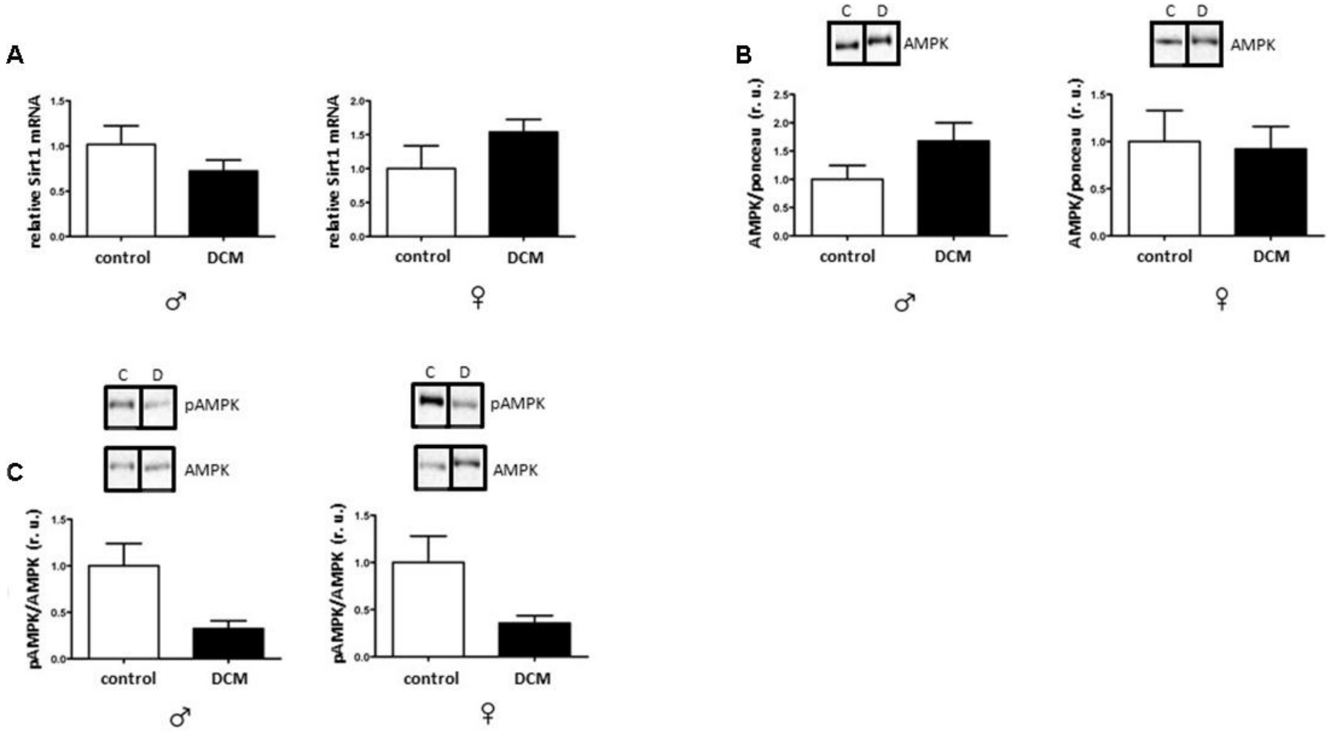

Supplementary Figure 1. DCM effects on Sirt1 expression and AMPK phosphorylation in young patients. Western blot expression analysis of Sirt1 (**A**), AMPK (**B**) and phosphorylated AMPK (Thr172) (**C**) performed with human cardiac tissue lysates from young control (non-diseased) or DCM men (♂) and women (♀). Proteins were normalized to actin or ponceau. Data are shown as the mean ± SEM (n= 5). Representative imaging of western blot analysis; the lanes were run in two gels. All data were normalized to the corresponding control and expressed in relative units (r.u.).

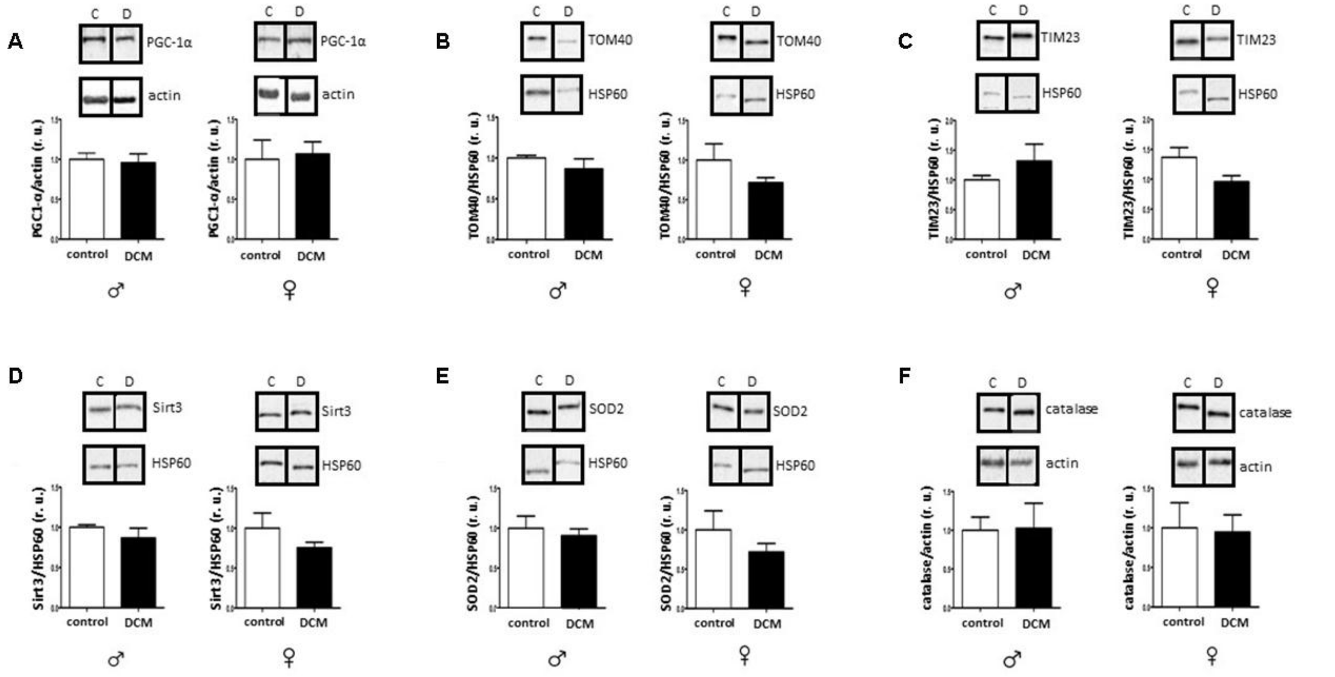

Supplementary Figure 2. DCM effects on expression of mitochondrial proteins in young patients. Western blot analysis and statistical analysis of PGC1-α (**A**), TOM40 (**B**), TIM23 (**C**), Sirt3 (**D**), SOD2 (**E**) and catalase (**F**) protein expression performed with human cardiac tissue lysates from young control (non-diseased) or DCM men (♂) and women (♀). Proteins were normalized to HSP60 or actin. Data are shown as the mean ± SEM (n= 5). Representative imaging of western blot analysis; the lanes were run in two gels. All data were normalized to the corresponding control and expressed in relative units (r.u.).

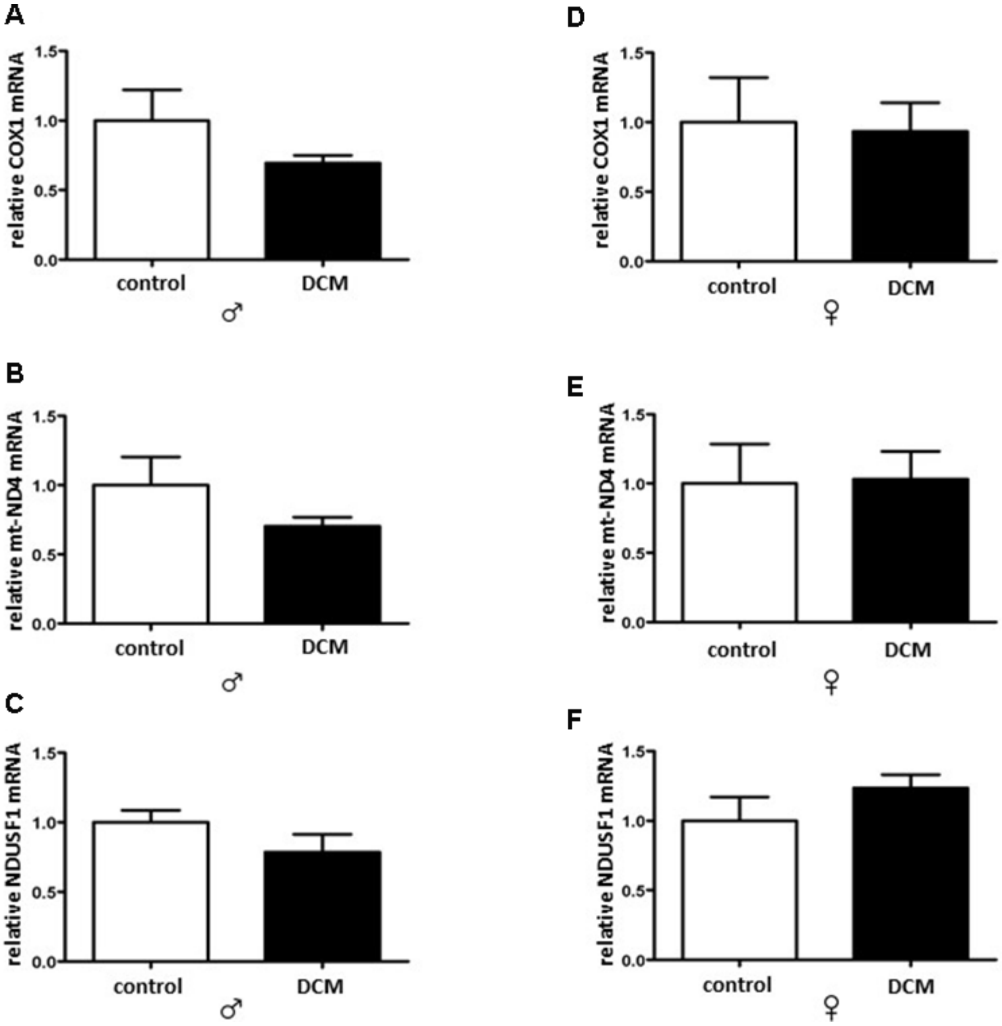

Supplementary Figure 3. DCM effects on expression of mitochondrial genes in young patients. Real-time PCR analysis for the mitochondrial-encoded genes *cox1* (**A**, **B**) and *mt-nda4* (**C**, **D**), as well as the nuclear-encoded genes *ndusf1* (**E**, **F**), performed with human cardiac tissue lysates from young control (non-diseased) or DCM men (♂) and women (♀). Data are shown as the mean ± SEM (n= 5).

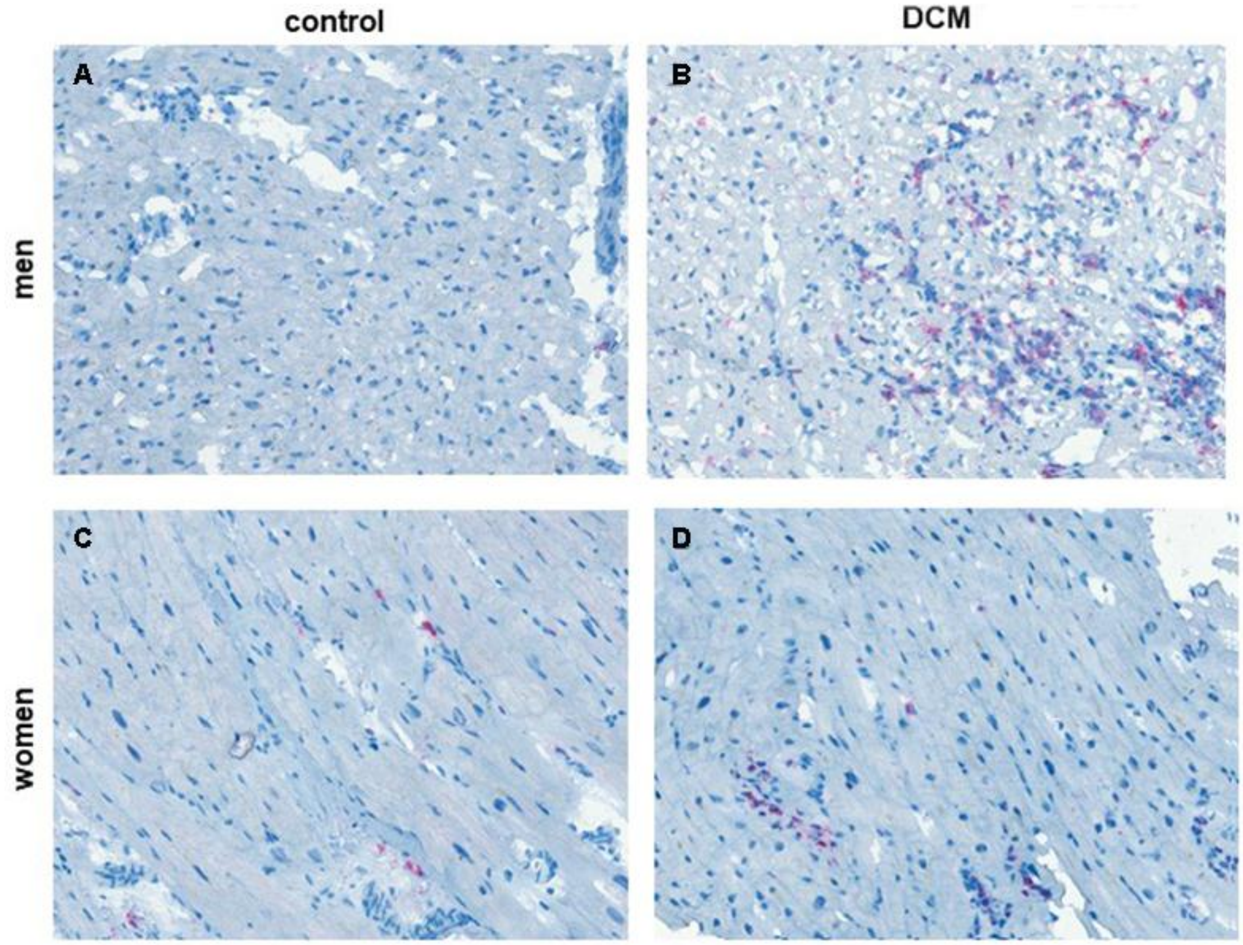

Supplementary Figure 4. DCM effects on the number of macrophages in cardiac tissue of DCM patients. Representative images of cardiac cryosections stained with antibodies against CD68 (**A**–**D**). The analyses were performed with myocardium from old control (non-diseased) and DCM men and women. Magnification: 200x.
